# Mental Imagery and Visual Working Memory

**DOI:** 10.1371/journal.pone.0029221

**Published:** 2011-12-14

**Authors:** Rebecca Keogh, Joel Pearson

**Affiliations:** School of Psychology, University of New South Wales, Sydney, Australia; National Institute of Mental Health, United States of America

## Abstract

Visual working memory provides an essential link between past and future events. Despite recent efforts, capacity limits, their genesis and the underlying neural structures of visual working memory remain unclear. Here we show that performance in visual working memory - but not iconic visual memory - can be predicted by the strength of mental imagery as assessed with binocular rivalry in a given individual. In addition, for individuals with strong imagery, modulating the background luminance diminished performance on visual working memory and imagery tasks, but not working memory for number strings. This suggests that luminance signals were disrupting sensory-based imagery mechanisms and not a general working memory system. Individuals with poor imagery still performed above chance in the visual working memory task, but their performance was not affected by the background luminance, suggesting a dichotomy in strategies for visual working memory: individuals with strong mental imagery rely on sensory-based imagery to support mnemonic performance, while those with poor imagery rely on different strategies. These findings could help reconcile current controversy regarding the mechanism and location of visual mnemonic storage.

## Introduction

The study of working memory has long been an area of interest for researchers due to its ubiquity in daily life, its close links to many high-level cognitive functions, psychopathologies [1] and the large individual variability present in both performance and capacity [2]–[4]. The storage mechanism and capacity limits of visual working memory have been and remain controversial [2], [5]–[8]. Likewise, the neural correlates of visual working memory have stirred up considerable debate, with some studies reporting sustained activity in high-level neural structures [9]–[11] while others, more recently, reporting early-level visual cortex [12], [13]. Behavioural studies support the involvement of early visual cortex, as they suggests that visual working memory can maintain visual information at a resolution typically only observed in early visual cortex [14]–[18].

There have been suggestions that visual working memory may involve mental imagery [19], [20], such propositions dovetail nicely with the visual spatial sketchpad component of composite theories of working memory [21], [22]. Interestingly the neural correlates of imagery have provoked a debate similar to the one in the visual working memory literature. Some neuroimaging studies have found no significant increase in neural activity in the early visual areas during imagery tasks [23]–[28]. More recently however, neuroimaging studies have found that early areas of the visual cortex are activated during imagery tasks as well as later visual areas [29]–[31]. Studies employing transcranial magnetic stimulation over early visual cortex further show that disruption of visual cortical activity can impair imagery tasks [32] and recent behavioural work has provided strong evidence that visual imagery is contingent on activity in early visual cortex [33], [34]. Interference style tasks also provide evidence that imagery may be involved in maintaining visual information in memory with some studies indicating that visual interference in the form of irrelevant pictures and dynamic visual noise deteriorates performance on both visual working memory and imagery tasks [20], [35]–[40]. However other work has failed to show these effects, with some studies finding no effects of dynamic visual noise on either working memory and/or imagery tasks [41]–[44].

Subjective reports of strategies employed during visual working memory may also provide insight into the role of imagery during visual working memory. Subjective reports from participants performing visual working memory tasks sometimes suggest a strategy that involves creating a detailed mental image to help performance [13], [45], [46]. These reports suggest that some participants may engage in the effortful generation of internal visual representations of the remembered items. The participant's descriptions are synonymous with definitions of mental imagery, potentially implicating imagery as a possible cognitive strategy used to solve visual working memory tasks.

Since the time of Sir Francis Galton [47] it has been noted that individuals differ in their self-reports of mental imagery ability. Some people report that they experience very intense, vivid images akin to actually seeing the item, whereas others report no ‘image’ *per se*, instead an individual's mental information seems to take on a more abstract, phonologically based feeling [48].

If large individual differences in both visual working memory and mental imagery are common [3], [29], [47], [49] and individuals report using imagery-like strategies during visual working memory tasks [13], [45], [46], and both involve activity in early visual cortex [13], [50], it follows that imagery may be an important cognitive element in working memory tasks.

However, studies examining the role of visual imagery in visual working memory tasks have produced mixed results [51]. Some studies have reported positive correlations [46], [52]–[54] whilst others have found no or negative relationships [51], [55], [56]. Despite this work, the exact nature of the relationship between visual imagery and working memory still remains unclear.

Here we capitalized on a new method to assess imagery, a visual phenomenon called binocular rivalry. This phenomenon involves presenting two different patterns, one to each eye, resulting in one pattern reaching awareness while the other is suppressed. A study by Pearson, Clifford & Tong (2008) found that when individuals imagined one of two rivalry patterns, that pattern had a higher probability of being dominant during a subsequent brief rivalry presentation. In fact, longer periods of imagery led to stronger bias effects, and these effects were highly specific to the orientation and location of the imagined pattern. Interestingly, when imagery was performed in the presence of a uniform illuminant background these effects tended to be weaker as a function of the background luminance [33], [57]. A recent study by Pearson, Rademaker & Tong (in press) has shown that subjective ratings of imagery vividness on a trial-by-trial basis predict the subsequent perceptual effect on binocular rivalry (but not on catch trials), while ratings of effort do not. Likewise off-line questionnaire ratings of imagery vividness tended to predict the strength of mental imagery as measured with binocular rivalry. This finding is important for the current work as it demonstrates that imagery as assessed using binocular rivalry is both a measure of its low-level sensory components and metacognitive sensations of vividness.

We utilized imagery's bias effect on subsequent binocular rivalry to investigate the role of imagery in different types of short-term visual memory (i.e. visual working memory and iconic memory). We show that individuals with strong imagery perform better in visual working memory tasks than individuals with poor imagery. However, imagery strength was unrelated to performance in iconic memory. In addition, we capitalized on the known ability of background luminance to interfere with imagery mechanisms to show that good imagers, but not poor imagers, tend to use imagery as a strategy for visual working memory tasks. This pattern only held for visual working memory and not for working memory of number strings, suggesting that luminance was attenuating sensory-based imagery and not general working memory mechanisms. These results provide compelling new evidence that imagery is a component of visual working memory for good imagers, whereas poor imagers likely rely on a different strategy. A dichotomy in cognitive strategies may help explain the diversity of results in visual working memory studies.

## Methods

### Participants

Thirty five undergraduate students (twenty female, aged 18–35) participated in the correlational study in exchange for course credit. All participants had normal or corrected to normal vision. All thirty participants completed each measure of memory and imagery for the first experiment. Seventeen undergraduate students (ten female, aged 18–44) participated in the causal luminance disruption experiments in exchange for course credit.

Informed written consent was obtained from all participants, and all experiments were approved by the UNSW Human Research Ethics Advisory Panel (Psychology).

### Apparatus

All of the tasks were performed in darkened rooms with black walls, to increase testing efficiency experiments were run on two calibrated computer monitors, one 19 inch Phillip Brilliance 109P4 monitor and a 21 inch Dell Trinitron monitor, both with a resolution of 1280×960 pixels, and a refresh rate of 75 Hz, one driven by a Mac Mini and the other an Imac computer. Experiments were run in Matlab, using Psychophysics toolbox [58], [59]. A fixed viewing distance of 57 cm for all experiments was obtained using a chinrest and participants were instructed to maintain fixation on the bull's-eye (a fixation point) at all times throughout the experiment, which acted as a fusion lock in the rivalry conditions. For the binocular rivalry experiment a mirror stereoscope was attached to the chin rest. The mirrors were carefully aligned for each individual participant so that the patterns from each eye overlapped to form visual rivalry.

### Stimuli

**Figure 1:**
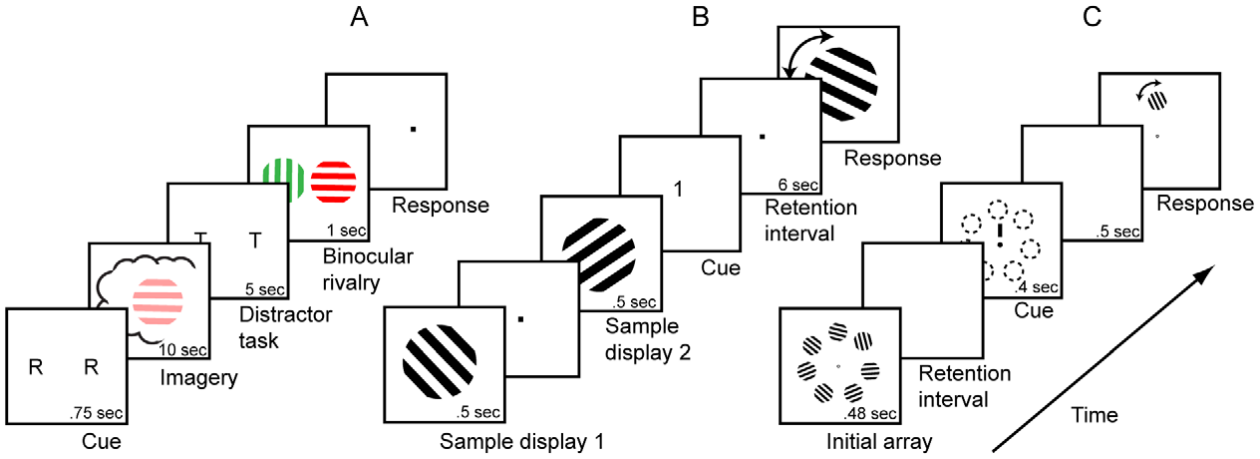
Experimental timelines and stimuli. (**A**)**.** During the imagery task participants were cued to imagine a red or green Gabor pattern for 10 sec. Next they performed a letter discrimination task, followed by the binocular rivalry display. Luminance profile shows the background luminance dynamics during the imagery period in experiment 2. (**B**)**.** For the visual working memory task participants were presented with two Gabor patterns sequentially, and were then cued to remember only one. Following a retention period a test pattern was presented and participants were required to indicate its orientation in relation to the pattern held in memory. (**C**)**.** During the iconic memory task participants were presented with seven Gabor patterns, and after the inter-stimulus interval (ISI) cued to remember only one of the seven. In the test phase a rotated Gabor pattern was displayed in the cued location and participants had to indicate its orientation in relation to the pattern held in memory.

In the imagery conditions the binocular rivalry stimuli consisted of red horizontal (CIE x = .277, y = 0.613) and green vertical (CIE x = 0.601, y = 0.368) Gabor patterns, 1 cycle/°, Gaussian *σ* = 3.5° (see figure 1A). The mean luminance of both Gabor patterns was 7.8 cdm^2^ (candela per square metre). Both patterns were presented in an annulus around a fixation spot. During the rapid serial visual presentation task, lower case letters (Times New Roman, 0.6° in height) were presented centrally. The background was black throughout the entire task, unless otherwise stated. For all luminance conditions the background ramped up to yellow (a mix of the green and red colors used for the rivalry patterns, with luminance at 7.8 cdm^ 2^) during the imagery period. During this period the background luminance was smoothly ramped up and down to avoid visual transients (see figure 1A).

The stimuli in the visual working memory task (for the correlational study) consisted of two Gabor patterns (1 cycle/°, Gaussian *σ* = 6°), 70% contrast (see figure 1B). The Gabor patterns were presented centrally and tilted clockwise at either 25° or 115°. The background throughout the entire task in the correlational study was grey with a luminance of 22 cdm^ −2^. The luminance experiments used green (CIE x = 0.601, y = 0.368) and red (CIE x = .277, y = 0.613) Gabor patterns, with all other pattern parameters held constant. The background was black for the duration of the experiment, with the exception of the retention interval during the luminance condition.

In the iconic memory task, seven grey Gabor patterns (1 cycle/°, Gaussian *σ* = 3°), 50% contrast, were used as the stimuli (see figure 1C). The seven Gabor patches evenly surrounded the bull's-eye fixation spot in a circular fashion, at a 10° diameter, so that they were somewhat in the participants' peripheral vision. Each of the seven Gabor patches had a unique orientation (between 0° to 360°) which was randomly selected for each trial. The background throughout the entire task was grey with a luminance of 22 cdm^ −2^.

All numbers in the number-string working memory experiment were white, presented on a black background (Times New Roman; 0.6° in height). During the retention interval the background was black for the no luminance condition and white in the luminance condition.

### Procedure

#### Binocular Rivalry

To control for individual differences in eye-dominance, which can lead to a perceptual bias for one eye, participants underwent an eye dominance test prior to the imagery task, as documented previously [33], [57].

During the imagery conditions observers were instructed to maintain fixation throughout each block of trials. For both the correlational and luminance condition, a central cue (“G” or “R”) was presented at the beginning of each trial to indicate whether participants should form a mental image of a green vertical grating or a red horizontal grating (see Figure 1A). This cue was randomized on each trial and appeared an equal number of times. Following the imagery period, participants underwent a distracter task in which they indicated when a “C” or “V” was present in the serial string of letters by pressing the corresponding keys (C or V) on the keyboard. Each letter was displayed for 300 ms. Participants then viewed the rivalry display for 750 ms and reported on the dominant pattern. Rivalry dominance was reported by pressing one of three assigned keys (1, 2 or 3) to indicate: (1) green vertical, (3) red horizontal, or for an approximately equal mixture of the two patterns (2) (due to binocular combination or piecemeal rivalry). To minimize potential response conflict, participants were required to use their left hand to complete the distracter task and their right hand for rivalry responses. Mixed mock rivalry trials were not used to calculate the bias measure of imagery as in previous work [33]. In the correlational study participants completed 40 binocular rivalry trials. Participants in the luminance manipulation experiments completed a total of 80 binocular rivalry trials, 40 with and 40 without a luminous background.

#### Iconic memory

Alpha-numeric figures are commonly used to assess iconic memory [60]. To avoid possible confounds from high-level cognitive information, the iconic memory task used Gabor patterns, which are difficult to encode phonologically. Hence, this should be a better measure of purely sensory visual memory. Participants held fixation while seven Gabor patterns (see figure 1C) were presented in the periphery in a circular fashion surrounding the fixation point. Following an inter-stimulus interval of 20 ms, 40 ms, or 400 ms a cue (line pointing to the previous Gabor position) was then presented. The location of the cued Gabor patch was randomized on each trial. A different Gabor pattern subsequently appeared in the cued position. The orientation of the Gabor patch was rotated either 20° clockwise (for half of the trials) or anticlockwise (for half of the trials) compared to the original Gabor pattern. Participants were asked to indicate whether the Gabor patch had been rotated clockwise or anticlockwise by pressing the right or left arrow key respectively. A total of 84 trials were completed by each of the participants.

#### Visual working memory (VWM)

The working memory task used in this experiment was based on Harrison and Tong's (2009) working memory paradigm (see figure 1B). Participants were presented with two Gabor patterns in a randomized consecutive order. A cue was then presented - either the number 1 or 2- which prompted the participants to remember either the first (cue number 1) or second (cue number 2) pattern. A retention interval of six seconds followed. After this interval a test pattern was presented rotated either +5° (for half of the trials) or -5° (for half of the trials) as compared to the remembered pattern and participants had to signal whether the test stimulus was rotated clockwise or anticlockwise relative to the one held in memory, by pressing the number 2 or 1 on the keypad respectively. Participants in the correlational study completed 40 trials. Participants in the luminance manipulation experiment completed a total of 80 trials, 40 with and 40 without a luminous background. It is worth noting that in this task test stimuli of 30 and 120° the correct answer was always clockwise. While for test stimuli of 20 and 110° the correct answer was always counter-clockwise. Hence, such a task may not be ideal for studies using larger number of total trials, as the test stimulus response association may be learnt over time. As there were only a total of 10/20 trials for each of the test stimuli in the current study (depending on the experiment), without any performance feedback, in a randomized order, the likelihood that participants were learning 4 specific orientations and their associated response is very low. In addition, Performance on the visual working memory task the individuals with strong mental imagery are disrupted by a luminance change that *only* occurs during the retention period, eg. during the test stimulus there is no luminance change. Hence, for individuals with strong mental imagery, performance on the visual working memory task has to be contingent on a process during the retention period.

#### Number string working memory (NWM)

The timing here was identical to the visual working memory task (see Figure 1B), however instead the Gabor pattern two strings of five numbers were presented sequentially. A cue was then presented, either the number 1 or 2, which prompted the participants to remember either the first (cue number 1) or second (cue number 2) number string. At the test phase participants were shown a 5 digit number string and were then asked to indicate whether the number was the same or different to the number they were cued to remember by pressing the number 1 or 2 on the keypad, respectively. Participants in the luminance manipulation experiment completed a total of 80 trials, 40 in the luminance condition and 40 in the no luminance condition.

## Results

**Figure 2:**
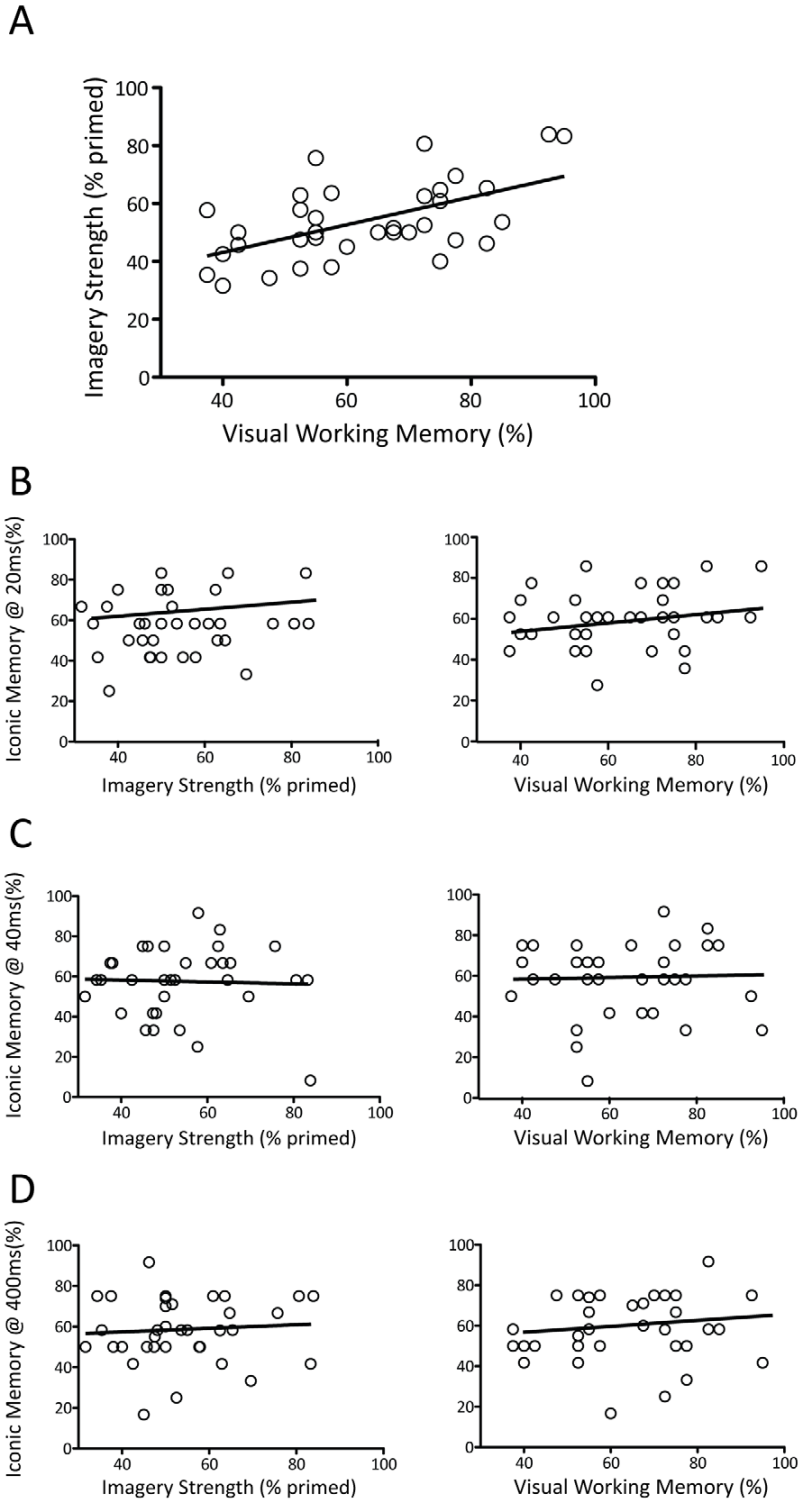
Correlational Results. (**A**)**.** Imagery ability was predictive of visual working memory performance. Each dot shows an individual participant. The trend line shows a linear fit to the data. (**B**)**.** There was no significant relationship between imagery strength and iconic memory performance for the 20 ms iconic memory ISI (left panel). Likewise, there was no significant correlation between visual working memory and iconic memory at 20 ms (right panel) (**C**)**.** Similarly there was no significant relationship between imagery strength and iconic memory performance at an ISI of 40 ms (left panel) or between iconic memory (40 ms) and visual working memory (right panel). (**D**)**.** There were no significant correlations between imagery strength (left panel) or visual working memory (right panel) for the iconic memory ISI of 400 ms. (For all correlations N = 34). Assumptions of normality, kurtosis, skewness and homoscadacity were met for all variables.

Figure 2A shows a scatter plot for visual working memory and mental imagery (as measured with binocular rivalry: see methods), each dot represents an individual participant (N = 35). There is a significant positive correlation between visual working memory and visual mental imagery, r(34) = .56, p<.001. That is, participants whose imagery had a strong bias effect on perceptual rivalry also tended to have more accurate visual working memory.

A letter discrimination task was added as part of the imagery procedure, after the imagery period and before the rivalry presentation. This task was included to prevent participants from continuing to imagine during the actual rivalry presentation, as concurrent imagery and perception could induce possible confounds of visual attention [61]. In addition, this task can be used to insure the pattern of data in the imagery task was not due to complacency or lack of effort. There was no significant relationship between the letter task performance and imagery ability (r(34) = . 07, p = .76). These results, taken together with previous studies using catch trials [33], [34], suggest that poor scores on the imagery task reflect a deficiency in imagery abilities and not complacent participants.

The relationship between imagery and iconic memory was then examined to assess whether imagery might be related to different types of visual memory. To assess iconic memory we used a circular array consisting of seven Gabor patterns (Figure 1C). In the iconic memory literature there are often variations in the different inter stimulus intervals used, likewise estimates of the longevity of iconic memory range from 20 to 500 ms (Sperling, 1960; Coltheart, 1980; Graziano & Sigman). For this reasons we used both short (20 ms and 40 ms) and long (400 ms) ISI durations. Across the long and short ISIs there were no significant correlations between iconic memory and mental imagery or between iconic memory and working memory ability. The left panels of Figures 2B, C and D show a non-significant correlation for the same participants between mental imagery and the iconic memory at 20, 40 and 400 ms (r(34) = .17, p = .34; r(34) = .13, p = .46; r(34) = .08, p = .67, respectively). These r values were all significantly different from the r value for visual working memory and imagery strength (Williams, t-test between non-independent Rs for: 20 ms t(31) = 2.07, p<0.05; 40 ms t(31) = 1.98, p<0.05; 400 ms t(31) = 2.42, p<0.05). Here, individuals with strong imagery tended to perform no better on the iconic memory task than individuals with poor mental imagery.

In addition, the relationship between visual working memory and iconic memory was examined to assess whether participants who performed well on one measure of visual memory also performed well on another. The right panels of figures 2B, C and D show non-significant relationships between iconic memory at 20, 40 and 400 ms and visual working memory (r(34) = .23, p = .19, r(34) = .04, p = .84, r(34) = .15, p = .41). All 3 r values were significantly different from the r value for visual working memory and imagery strength (Williams, t-test between non-independent Rs, all Ps<.05). This indicates that participants' performance on visual working memory is not indicative of their performance on iconic memory.

To further investigate the role of mental imagery in visual working memory we manipulated the luminance of the background during both the working memory and imagery tasks (see Figures 1A & B). Previous studies have demonstrated that greater levels of background luminance lead to an attenuation of imagery [33] and this is not due to dark adaptation [57]. It follows that if all our participants were using imagery to solve the visual working memory task, altering background luminance should attenuate the imagery mechanism and hence also affect visual working memory performance. However, if only some participants were adopting a strategy that utilized imagery to solve the visual working memory task, we might expect a decline in memory performance in only a subset of participants. To assess whether luminance signals might attenuate not only imagery processes but also general mechanisms of working memory, we included a non-spatial ‘higher-level’ working memory task in which participants were required to remember a number string instead of a visual pattern (see methods).

**Figure 3:**
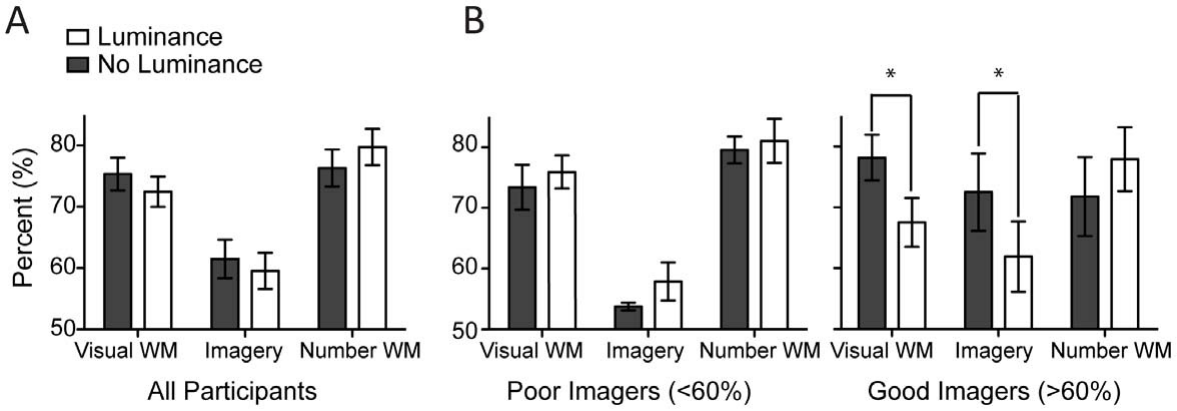
Luminance manipulation experiments. Luminance attenuates working memory and imagery, but only for good imagers. (**A**)**.** The graph shows the mean performance for all participants in the luminance and no luminance conditions. There is no significant effect of luminance. Error bars show ± SEMs (N = 17). (**B**)**.** Data separated by imagery ability using a median split. The leftmost graph shows the data for poor imagers (N = 10), graph on the right for good imagers (N = 7), error bars show ± SEM. Good imagers show an effect of background luminance. It should be noted that separating participants by imagery ability using a mean split resulted in the same patterns with no differences in performance for poor imagers and attenuation effects for good imagers when in the luminance condition for visual working memory and luminance respectively, t(5) =  −6.19, p = .002, and t(5) =  −4.21 p = .008.

Figure 3A displays the mean scores for all participants for both the luminance and no luminance conditions. There is a weak trend for luminance to attenuate imagery and visual working memory performance. However, this effect did not reach significance for either imagery or visual working memory (t(16) =  −.75, p = .47; t(16) =  −.98, p = .34; respectively). Surprisingly the presence of luminance tended to facilitate performance on the number-string working memory task, with participants performing better in the luminance condition (79%) than in the no luminance condition (76%). However, this difference was also not significant, t(16) = 1.29, p = .22

We hypothesized that perhaps only a subset of participants might be using a strategy that involved imagery. Perhaps individuals with strong imagery were more likely to use mental images to boost mnemonic performance. To test this we split the data into two groups (a median split at ∼60% in the imagery task) based on each participant's individual imagery score (the ‘good’ imagers group had significantly more perceptual bias in the no luminance condition than the ‘poor’ imagers, t(15) = 4.18, p = 0.001). This resulted in 10 individuals being placed in the poor imagery group and 7 in the good imagery group.

Figure 3B shows the mean scores for the poor imagery group on the left and the good imagery group on the right. Interestingly, on average poor imagers performed slightly better in the presence of luminance for all tasks. However this trend was not significant for visual working memory (t(9) = .50), number-string (t(9) =  −.46, p = .66), or imagery (t(9) = 1.0).

The right side of figure 3B shows data from the good imagers for the three conditions, with and without luminance. The presence of luminance attenuated both visual working memory and imagery performance, with participants performing significantly better in the no luminance condition in comparison to the luminance condition for both visual working memory and imagery, t(6) =  −3.79, p = .009, and t(6) =  −4.77 p = .003, respectively. For good imagers we found that performance in the number-string task was slightly better in the luminance condition, however this trend was not significant (t(6) = 1.73 p = .13).

## Discussion

Our study indicates a positive correlation between imagery and visual working memory performance. No such relationships were found between imagery and iconic memory, or iconic memory and visual working memory. For individuals with strong mental imagery, luminance attenuated performance in visual working memory and imagery tasks, but did not affect memory for number-strings. This suggests that those who have strong imagery may utilise it to aid performance in visual working memory tasks. We have demonstrated that having strong mental imagery is an asset in regards to solving visual working memory tasks, however being a good imager does not seem to have any bearing on performance on other forms of visual memory, such as iconic memory.

Our results suggest that individuals might use different cognitive strategies to solve the same visual memory task. More specifically good imagers might use imagery to solve the memory task. Poor imagers on the other hand do not, or are not able to create visual images to a useful degree. However, individuals with poor imagery still performed well in both working memory tasks. This suggests that poor imagers likely rely on a non-imagery based strategy perhaps a more ‘language like’ verbal workspace to complete the task, using semantic propositional information from the mnemonic stimuli, much like the strategy employed to store the number-string information [48].

The current work also suggests that irrelevant visual stimuli can interfere with the visio-spatial sketchpad, however this interference might only occur when participants use imagery as a strategy in solving visual working memory tasks. Current studies investigating the effects of irrelevant visual stimuli on visual working memory tasks have been working under the assumption that all people process and manipulate visual stimuli in the same manner. However, if, as our results suggest, only good imagers use visual imagery to solve visual working memory tasks it might not come as a surprise that some studies have found interfering effects from visual distractors and others have not.

Despite the correlation between visual working memory and mental imagery in experiment 1, participants with poor imagery could still perform the visual working memory task (e.g. fig. 3B). In other words, individuals with perhaps almost no functional mental imagery could still perform well above chance in the visual working memory task. This suggests that individuals were not utilizing visual working memory skills to perform mental imagery, but the other way around. If subjects were using visual working memory mechanisms to perform imagery, we might expect the degree of poor imagery to be limited by working memory performance, however we demonstrate that imagery can almost be functionally non-existent (according to our measure), while visual working memory performance remains reasonable. Hence, we propose that imagery might be an element in a compound working memory system, such as the proposed visual spatial sketchpad [21], [62].

There is now strong evidence that early visual areas are recruited and used during mental imagery tasks [33], [63]. If early visual areas are required to create detailed mental images [64] and, as our results suggest, only good imagers use a mental imagery strategy when solving visual working memory tasks, it is possible that poor and good imagers recruit different neural substrates when performing visual working memory tasks. If a propositional or ‘language like’ strategy was employed, high-level semantic and symbolic brain regions might be recruited as opposed to early visual areas. Conversely, if an imagery strategy were used, we would expect to see activation in early visual areas. If there is such a dichotomy in strategies, our results may help explain the current inconsistency in the literature in regards to the neural correlates of visual working memory [9]–[13], [65], [66]. Some previous studies have failed to take into account individual differences in either imagery or working memory performance. If individuals do use different cognitive strategies to solve visual working memory tasks, which in turn use different neural structures, this might explain why some studies have found increased BOLD in early visual areas, while others have not. The investigation of such individual differences may therefore provide a valuable contribution to theoretical models of working memory.

The use of different cognitive strategies to complete visual working memory tasks may also provide some insight into current theories of working memory capacity. There are two primary theories concerning the capacity and storage mechanisms of visual working memory: the discrete resource model [7], [67], [68] and the flexible resource model [5], [69]. The discrete resource model proposes that there is a limited number of ‘slots’ in memory that can each be occupied with a single item, whereas the flexible resource model posits that a finite memory resource can be spread out over many sensory items at differing degrees of precision [2], [7], [69]. Substantial evidence for and against both models exists. However, neither theory is largely influenced by individual differences in memory capacity, even though such individual differences are well documented [2], [3], [49]. With individual differences in visual working memory capacity ranging from 1 item to 7 items [2], [7], [49], [66], [70], [71], it is somewhat surprising that current models do not factor in potential causes behind such differences. If stronger imagery does in fact boost performance accuracy in visual working memory tasks, it may similarly have a modulatory effect on capacity limits. If this is the case, one may expect to find large individual differences in capacity limits that parallel the documented individual differences in imagery strength.

Behavioural work has shown that imagery can alter sensory perception [33], [57], [72], [73]. If imagery is in fact utilized during visual working memory then one might expect the contents of visual working memory to likewise alter sensory perception. This is exactly what has recently been found [15]. Here the authors report that the content of visual working memory directly changed perception of a separate visual stimulus.

It will be interesting for future studies to assess the impact of individual differences and even to incorporate the known characteristics of imagery into theoretical models of visual working memory. Our results suggest that individuals with strong imagery will utilize it during visual working memory tasks and that this may give them a competitive edge, allowing for greater mnemonic accuracy. Future work should shed light on the physiological basis of stronger and more vivid imagery, while unlocking the intricate relationship between imagery and many cognitive and sensory functions.

## References

[1] Park S, Gibson C, McMichael T (2006). Socioaffective factors modulate working memory in schizophrenia patients.. Neuroscience.

[2] Fukuda K, Awh E, Vogel EK (2010). Discrete capacity limits in visual working memory.. Curr Opin Neurobiol.

[3] Vogel EK, Machizawa MG (2004). Neural activity predicts individual differences in visual working memory capacity.. Nature.

[4] Zimmer HD (2008). Visual and spatial working memory: from boxes to networks.. Neurosci Biobehav Rev.

[5] Bays PM, Catalao RF, Husain M (2009). The precision of visual working memory is set by allocation of a shared resource.. J Vis.

[6] Edin F, Klingberg T, Johansson P, McNab F, Tegner J (2009). Mechanism for top-down control of working memory capacity.. Proc Natl Acad Sci U S A.

[7] Rouder JN, Morey RD, Cowan N, Zwilling CE, Morey CC (2008). An assessment of fixed-capacity models of visual working memory.. Proc Natl Acad Sci U S A.

[8] Serences JT, Saproo S (2010). Population Response Profiles in Early Visual Cortex Are Biased in Favor of More Valuable Stimuli.. Journal of Neurophysiology.

[9] Cornette L, Dupont P, Salmon E, Orban GA (2001). The neural substrate of orientation working memory.. J Cogn Neurosci.

[10] Fuster JM, Bauer RH, Jervey JP (1981). Effects of cooling inferotemporal cortex on performance of visual memory tasks.. Exp Neurol.

[11] Smith EE, Jonides J (1999). Storage and executive processes in the frontal lobes.. Science.

[12] Ester EF, Serences JT, Awh E (2009). Spatially global representations in human primary visual cortex during working memory maintenance.. J Neurosci.

[13] Harrison SA, Tong F (2009). Decoding reveals the contents of visual working memory in early visual areas.. Nature.

[14] Pasternak T, Greenlee MW (2005). Working memory in primate sensory systems.. Nat Rev Neurosci.

[15] Kang MS, Hong SW, Blake R, Woodman GF (2011). Visual working memory contaminates perception.. Psychonomic Bulletin & Review.

[16] Magnussen S (2000). Low-level memory processes in vision.. Trends in neurosciences.

[17] Magnussen S, Greenlee MW, Asplund R, Dyrnes S (1991). Stimulus-specific mechanisms of visual short-term memory.. Vision research.

[18] Magnussen S, Idas E, Myhre SH (1998). Representation of orientation and spatial frequency in perception and memory: a choice reaction-time analysis.Journal of experimental psychology Human perception and performance.

[19] Bruyer R, Scailquin JC (1998). The visuospatial sketchpad for mental images: Testing the multicomponent model of working memory.. Acta Psychologica.

[20] Baddeley, Andrade J (2000). Working memory and the vividness of imagery.. J Exp Psychol Gen.

[21] Logie R (1995). Visio-spatial working memory..

[22] Baddeley AD (1992). Working memory.. Science.

[23] D'Esposito M, Detre JA, Aguirre GK, Stallcup M, Alsop DC (1997). A functional MRI study of mental image generation.. Neuropsychologia.

[24] Kaas A, Weigelt S, Roebroeck A, Kohler A, Muckli L (2010). Imagery of a moving object: the role of occipital cortex and human MT/V5+.. NeuroImage.

[25] Mazard A, Tzourio-Mazoyer N, Crivello F, Mazoyer B, Mellet E (2004). A PET meta-analysis of object and spatial mental imagery.. European Journal of Cognitive Psychology.

[26] Mellet E, Petit L, Mazoyer B, Denis M, Tzourio N (1998). Reopening the mental imagery debate: Lessons from functional anatomy.. Neuroimage.

[27] Mellet E, Tzourio N, Denis M, Mazoyer B (1995). A Positron Emission Tomography Study of Visual and Mental Spatial Exploration.. Journal of Cognitive Neuroscience.

[28] Mellet E, Tzourio N, Denis M, Mazoyer B (1998). Cortical anatomy of mental imagery of concrete nouns based on their dictionary definition.. Neuroreport.

[29] Cui X, Jeter CB, Yang D, Montague PR, Eagleman DM (2007). Vividness of mental imagery: individual variability can be measured objectively.. Vision Res.

[30] Kosslyn SM, Alpert NM, Thompson WL (1997). Neural systems that underlie visual imagery and visual perception: A PET study.. Journal of Nuclear Medicine.

[31] Kosslyn SM, Alpert NM, Thompson WL, Maljkovic V, Weise SB (1993). Visual Mental-Imagery Activates Topographically Organized Visual-Cortex - Pet Investigations.. Journal of Cognitive Neuroscience.

[32] Kosslyn SM, Pascual-Leone A, Felician O, Camposano S, Keenan JP (1999). The role of area 17 in visual imagery: convergent evidence from PET and rTMS.. Science.

[33] Pearson J, Clifford CWG, Tong F (2008). The functional impact of mental imagery on conscious perception.. Current Biology.

[34] Pearson J, Rademaker R, Tong F Evaluating the mind's eye: The metacognition of visual imagery.. Psychological Science.

[35] Darling S, Della Sala S, Logie RH (2007). Behavioural evidence for separating components within visuo-spatial working memory.. Cognitive processing.

[36] Dean GM, Dewhurst SA, Morris PE, Whittaker A (2005). Selective interference with the use of visual images in the symbolic distance paradigm.Journal of experimental psychology Learning, memory, and cognition.

[37] Dean GM, Dewhurst SA, Whittaker A (2008). Dynamic visual noise interferes with storage in visual working memory.. Experimental psychology.

[38] Dent K (2010). Dynamic visual noise affects visual short-term memory for surface color, but not spatial location.. Experimental psychology.

[39] McConnell J, Quinn JG (2000). Interference in visual working memory.The Quarterly journal of experimental psychology A, Human experimental psychology.

[40] Wais PE, Rubens MT, Boccanfuso J, Gazzaley A (2010). Neural mechanisms underlying the impact of visual distraction on retrieval of long-term memory.. The Journal of neuroscience.

[41] Andrade J, Kemps E, Werniers Y, May J, Szmalec A (2002). Insensitivity of visual short-term memory to irrelevant visual information.The Quarterly journal of experimental psychology A, Human experimental psychology.

[42] Zimmer HD, Speiser HR (2002). The irrelevant picture effecting visio-spatial working memory: fact or fiction?. Psychological Beitrage.

[43] Avons SE, Carlo s (2005). Dynamic visual noise: No interference with visual short-term memory or the construction of visual images.. The European Journal of Cognitive Psychology.

[44] Pearson DG, Logie RH, Gilhooly KJ (1999). Verbal representations and spatial manipulation during mental synthesis.. European Journal of Cognitive Psychology.

[45] Berger GH, Gaunitz SC (1979). Self-rated imagery and encoding strategies in visual memory.. Br J Psychol.

[46] Gur RC, Hilgard ER (1975). Visual imagery and the discrimination of differences between altered pictures simultaneously and successively presented.. Br J Psychol.

[47] Galton F (1880). Statistics of mental imagery.. Mind.

[48] Pylyshyn ZW (2003). Return of the mental image: are there really pictures in the brain?. Trends Cogn Sci.

[49] Vogel EK, Awh E (2008). How to exploit diversity for scientific gain: Using individual differences to constrain cognitive theory.. Current Directions in Psychological Science.

[50] Slotnick SD (2008). Imagery: Mental pictures disrupt perceptual rivalry.. Current Biology.

[51] Reisberg D, Leak S (1987). Visual imagery and memory for appearance: does Clark Gable or George C. Scott have bushier eyebrows?. Can J Psychol.

[52] Hanggi D, Steiner GF (1989). Individual-Differences in Visual-Imagery.. Bulletin of the Psychonomic Society.

[53] Marks DF (1973). Visual imagery differences in the recall of pictures.. Br J Psychol.

[54] Baddeley AD, Andrade J (2000). Working memory and the vividness of imagery.. J Exp Psychol Gen.

[55] Heuer F, Fischman D, Reisberg D (1986). Why does vivid imagery hurt colour memory?. Can J Psychol.

[56] Reisberg D, Culver LC, Heuer F, Fischman D (1986). Visual memory: When imagery vividness makes a difference.. Journal of Mental Imagery.

[57] Sherwood R, Pearson J (2010). Closing the mind's eye: incoming luminance signals disrupt visual imagery.. PLoS One.

[58] Brainard DH (1997). The Psychophysics Toolbox.. Spatial vision.

[59] Pelli DG (1997). The VideoToolbox software for visual psychophysics: transforming numbers into movies.. Spatial vision.

[60] Long GM (1980). Iconic memory: a review and critique of the study of short-term visual storage.. Psychological bulletin.

[61] Carrasco M, Ling S, Read S (2004). Attention alters appearance.. Nat Neurosci.

[62] Baddeley AD, Hitch GJ (1974). Working Memory..

[63] Kosslyn SM, Ganis G, Thompson WL (2001). Neural foundations of imagery.. Nat Rev Neurosci.

[64] Kosslyn SM, Thompson WL (2003). When is early visual cortex activated during visual mental imagery?. Psychological Bulletin.

[65] Offen S, Schuppeck D, Heeger DJ (2009). The role of early visual cortex in visual short-term memory and visual attention.. Vision Research.

[66] Todd JJ, Marois R (2005). Posterior parietal cortex activity predicts individual differences in visual short-term memory capacity.. Cogn Affect Behav Neurosci.

[67] Johnson JS, Spencer JP, Luck SJ, Schoner G (2009). A dynamic neural field model of visual working memory and change detection.. Psychol Sci.

[68] Zhang WW, Luck SJ (2008). Discrete fixed-resolution representations in visual working memory.. Nature.

[69] Bays PM, Husain M (2008). Dynamic shifts of limited working memory resources in human vision.. Science.

[70] Unsworth N, Engle RW (2007). The nature of individual differences in working memory capacity: active maintenance in primary memory and controlled search from secondary memory.. Psychol Rev.

[71] Vogel EK, McCollough AW, Machizawa MG (2005). Neural measures reveal individual differences in controlling access to working memory.. Nature.

[72] Zimmer HD, Speiser HR, Seidler B (2003). Spatio-temporal working-memory and short-term object-location tasks use different memory mechanisms.. Acta Psychologica.

[73] Mohr HM, Linder NS, Dennis H, Sireteanu R (2011). Orientation-specific aftereffects to mentally generated lines.. Perception.

